# Predictive factors on CT imaging for progression of uncomplicated into complicated acute diverticulitis

**DOI:** 10.1007/s00384-017-2919-0

**Published:** 2017-10-26

**Authors:** S. T. van Dijk, L. Daniels, C. Y. Nio, I. Somers, A. A. W. van Geloven, M. A. Boermeester

**Affiliations:** 1 Department of Surgery, Academic Medical Center, Meibergdreef 9, 1100 DD, PO Box 22660, Amsterdam, The Netherlands; 2 Department of Radiology, Academic Medical Center, Amsterdam, The Netherlands; 3 Department of Surgery, Tergooi Hospital, Hilversum, The Netherlands

**Keywords:** Acute diverticulitis, CT imaging, Disease progression, Uncomplicated, Complicated

## Abstract

**Purpose:**

Since outpatient treatment and omitting antibiotics for uncomplicated acute colonic diverticulitis have been proven to be safe in the majority of patients, selection of patients that may not be suited for this treatment strategy becomes an important topic. The aim of this study is to identify computed tomography (CT) imaging predictors for a complicated disease course of initially uncomplicated acute diverticulitis.

**Methods:**

CT imaging from a randomized controlled trial (DIABOLO study) of an observational vs. antibiotic treatment strategy of first-episode uncomplicated acute diverticulitis patients was re-evaluated. For each patient that developed complicated diverticulitis within 90 days after randomization, two patients with an uncomplicated disease course were randomly selected. Two abdominal radiologists, blinded for outcomes, independently re-evaluated all CTs.

**Results:**

Of the 528 patients in the DIABOLO trial, 16 patients developed complications (abscess > 5 cm, perforation, bowel obstruction) within 90 days after randomization. In the group with a complicated course of initially uncomplicated diverticulitis, more patients with fluid collections (25 vs. 0%; *p* = 0.009) and a longer inflamed colon segment (86 ± 26 mm vs. 65 ± 21 mm; *p* = 0.007) were observed compared to an uncomplicated course of disease. Pericolic extraluminal air was no predictive factor.

**Conclusion:**

Fluid collections and to a lesser extent the length of the inflamed colon segment may serve as predictive factors on initial CT for a complicated disease course in patients with uncomplicated acute colonic diverticulitis. These findings may aid in the selection of patients not suitable for outpatient treatment and treatment without antibiotics.

## Introduction

From all patients with acute colonic diverticulitis, roughly two-third presents with uncomplicated diverticulitis [1]. Traditionally, these uncomplicated patients were admitted to the hospital and antibiotic treatment was initiated routinely. Meanwhile, two randomized controlled trials showed that antibiotics can safely be omitted in the treatment of uncomplicated acute diverticulitis [2, 3]. Furthermore, a recent systematic review showed that outpatient treatment of uncomplicated diverticulitis is safe, effective, and economically efficient in a selected group of patients [4].

An important cause of failure of both omitting antibiotics and outpatient treatment is progression of an uncomplicated episode of diverticulitis into a complicated diverticulitis episode [5, 6]. Currently, computed tomography (CT) is only used to establish the diagnosis and stage of disease at presentation, whereas some clinical characteristics are used to predict the course of disease after presentation. Clinical judgment is used to select patients that may not be suitable for outpatient treatment and omitting antibiotics.

Identification of predictive factors on CT imaging could improve patient selection for more aggressive treatment than simple observation and possibly prevent progression of disease into complicated diverticulitis or ameliorate its course. The aim of this study was to identify those predictive factors using CT imaging and patient outcomes from the DIABOLO trial, a randomized controlled trial on observational vs. antibiotic treatment in patients with CT-proven uncomplicated acute diverticulitis.

## Methods

### Study design and patient population

The DIABOLO trial was a randomized controlled trial, taking place in 22 clinical sites in The Netherlands during 2010–2012 [3]. A total of 528 patients with CT-proven, first-episode, left-sided, and uncomplicated acute diverticulitis were randomized to either an observational (262 patients) or an antibiotic (266 patients) treatment strategy. Uncomplicated acute diverticulitis was defined as modified Hinchey stages 1a and 1b [7]; therefore, patients having a small pericolic abscess (< 5 cm) or solely pericolic free air with absence of ascites or abscess were also included in the study.

In the present study, all patients that developed complicated disease course of uncomplicated diverticulitis within 90 days after randomization were identified. Complicated diverticulitis within these 90 days was considered an escalation of the initial uncomplicated episode. Complicated diverticulitis after these 90 days was considered to be a new episode of acute diverticulitis and therefore not directly related to the initial CT at the time of randomization.

Subsequently, for each complicated case, two cases were selected from the group of patients that did not develop complicated diverticulitis. These uncomplicated cases were analyzed as controls. To account for the diversity in CT scanners and CT protocols between the different hospitals, the two uncomplicated cases were randomly selected (using random sampling in SPSS) from the same hospital as the complicated case.

### Data collection and outcomes

CT imaging at the time of randomization was obtained from the participating hospital for each selected patient. Two abdominal radiologists (CN and IS with respectively 20 and 10 years of experience), both from a tertiary academic center, re-evaluated each CT. Both were blinded for patient characteristics, initial CT report from the participating hospital, CT report from the other expert reader, and patient outcome. Consequently, both radiologists were unaware if the CT was from the complicated group or control group. They only knew some patients had a deviant clinical course but were unaware of the proportion of patients. A case record form was used to collect the CT data. Re-evaluation and all measurements were performed in Agfa-IMPAX Version 6.5 software.

**Fig. 1:**
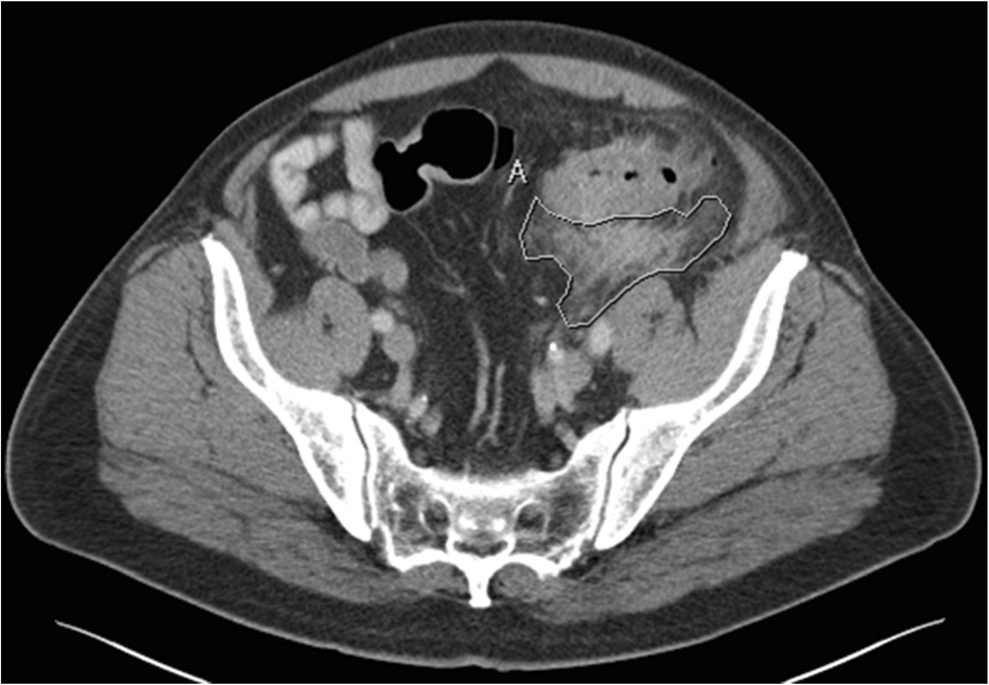
CT slide showing pericolic inflammation secondary to acute diverticulitis. Pericolic inflammation is measured using a mean region of interest (ROI) resulting in an area of inflammation of 23.65 cm^2^ with a mean Hounsfield unit of + 17.40

The CT-scan characteristics that were registered were tube kilovoltage, tube current, slice thickness, type of multiplanar reconstructions used, image quality, and the use of intravenous, oral, and rectal contrast. CT outcome measures were the presence and location of extraluminal air (pericolic or distant), free fluid (fluid that is not walled off), and fluid collections (fluid that is walled off, with or without enhancing wall or entrapped gas); presence, location, maximum size, and number of colonic diverticula; location and length of the inflamed colon segment; presence and area of pericolic inflammation (increased density of pericolic fat tissue); maximal colonic wall thickness; presence of an enhancing colonic wall; and the presence of enlarged lymph nodes. For the outcome measure fluid collections, distinction was made between collection with or without typical characteristics for an abscess, namely an enhancing wall and entrapped air. The largest diameter of the fluid collection was measured in the axial plane. Pericolic inflammation was measured using a mean region of interest (ROI) measurement in the axial plane image containing maximal inflammation. This measurement resulted in the area of inflammation in square centimeters and mean Hounsfield unit (Fig. 1). Length of the inflamed colon segment was measured perpendicular to the luminal axis.

### Statistical analysis

For dichotomous outcomes, agreement between the two radiologists was denoted by reporting the number of patients designated for each outcome in two categories. The category “consensus” consists of patients that both radiologists independently agreed upon, the category “no consensus” consists of patients that were designated by only one radiologist. Only the category “consensus” was statistically compared, as these findings were most reliable. Interrater reliability was also assessed by calculating Cohen’s kappa (ƙ) for categorical variables and intra-class correlation coefficient (ICC) for continuous variables. A Cohen’s kappa or ICC of less than 0.20 represents slight agreement, 0.21–0.40 represents fair agreement, 0.41–0.60 moderate agreement, 0.61–0.80 substantial agreement, and above 0.81 represents almost perfect agreement. For continuous outcomes, the mean of the values from the two radiologists was calculated before calculating the mean or median for the whole group. Categorical outcomes were compared using the chi-square test of Fisher’s exact test, as appropriate. Continuous outcomes were compared using the unpaired *t* test. A *p* value < 0.05 was considered statistically significant. All analyses were performed using SPSS, version 23.0 (SPSS Inc., Chicago, IL, USA).

## Results

Of the 528 patients with uncomplicated diverticulitis in the DIABOLO trial, 16 patients progressed into complicated diverticulitis within 90 days after randomization. Four patients developed an abscess larger than 5 cm, six patients developed perforation, and six patients developed bowel obstruction demanding surgical intervention. From those six patients with bowel obstruction, one patient also had a diverticular bleeding 18 days prior to the obstruction.

Initial CT imaging of 16 initially uncomplicated patients that progressed to complicated diverticulitis and initial CT imaging of 32 patients that remained uncomplicated was re-evaluated and compared. CT imaging from 11 different hospitals was used for this study. CT-scan settings and characteristics were comparable between the two groups (Table 1). In the uncomplicated group, the oral contrast had reached a deeper level in the intestine than the complicated group; in 71 vs. 12% of patients, oral contrast had reached at least the descending colon.

**Table 1:** Comparison of CT-scan characteristics in uncomplicated acute diverticulitis patients remaining without complications vs. patients developing complications after an at first uncomplicated episode

	Without complications (*N* = 32)	Developed complications (*N* = 16)
Tube kilovoltage (kVp)^†^	120 (120–120)	120 (120–120)
Tube current milliampere second (mAs)^†^	165 (108–202)	177 (115–238)
Slice thickness (mm)^†^	5.00 (3.00–5.00)	4.00 (3.00–5.00)
Multiplanar reconstruction—no (%)		
Axial	32 (100%)	16 (100%)
Coronal	30 (94%)	12 (75%)
Sagittal	11 (34%)	4 (25%)
Image quality—no (%)		
Good	27 (84%)	14 (87%)
Moderate	5 (16%)	2 (13%)
Contrast—no (%)		
Intravenous	27 (84%)	15 (94%)
Oral	17 (53%)	9 (56%)
Deepest level reached		
Small intestine	3 (18%)	4 (44%)
Caecum/transverse colon	2 (12%)	4 (44%)
Descending colon/sigmoid	6 (35%)	0 (0%)
Rectum	6 (35%)	1 (12%)
Rectal	2 (6%)	0 (0%)

^†^Median and interquartile ranges

### Patients who developed complicated diverticulitis after initially uncomplicated diverticulitis vs. patients who remained uncomplicated

At re-evaluation, CT imaging of 13 patients showed extraluminal air: 25% in the uncomplicated group and 31% in the complicated group (*p* = 0.735). All were considered pericolic extraluminal air (Table 2). Distant air was seen in three patients, but radiologists did not agree on this observation. Free fluid was seen in 25% of patients in the group that progressed to complicated disease vs. 9% of patients in the uncomplicated group (*p* = 0.201). There was low agreement between the radiologists regarding the location of free fluid, but it frequently was located anterior of the rectum in both groups. Significantly more patients with fluid collections were seen in the complicated group (25 vs. 0% respectively, *p* = 0.009). All of those fluid collections were pericolic and three out of four met the criteria for evident abscess (entrapped air and enhancing wall).

**Table 2:** Comparison of radiological findings in uncomplicated acute diverticulitis patients remaining without complications vs. patients developing complications after an at first uncomplicated episode

	Without complications (N = 32)		Developed complications (N = 16)		P value Consensus comparison	Interrater reliability^#^
	Consensus	No consensus	Consensus	No consensus		
Extraluminal air present—no (%)	8 (25)	1 (3)	5 (31)	3 (19)	0.735	0.81
Location						
Pericolic	7	1	4	2		
Distant	0	1	0	2		
Free fluid present—no (%)	3 (9)	3 (9)	4 (25)	1 (6)	0.201	0.68
Location						
Pericolic	1	3	0	1		
Anterior of the rectum	0	4	3	3		
Paracolic/subphrenic	0	0	0	0		
Fluid collection present—no (%)	0 (0)	2 (6)	4 (25)	1 (6)	0.009	0.70
Location						
Pericolic	0	2	4	0		
Anterior of the rectum	0	0	0	1		
Paracolic/subphrenic	0	0	0	0		
Entrapped air	0	1	3	0		
Enhancing wall	0	1	3	0		
Largest axial diameter (mm)^‡^	30,0 ± 9,9		35,0 ± 9,9			
Colonic diverticula present—no (%)	31 (97)	1 (3)	16 (100)	0 (0)	1.000	N/A**
Location						
Only in inflamed segment—no (%)	0	8	3	1		
Total number in entire colon						
< 5	0	3	0	1		
5–10	2	9	2	3		
10–20	3	10	3	4		
> 20	13	5	6	2		
Diameter largest diverticulum (mm)^‡^	11.5 ± 2.3		10.2 ± 2.2		0.154	0.42^$^
Inflamed colon segment present—no (%)	32 (100)	0 (0)	16 (100)	0 (0)		
Location						
Descending colon	7	6	0	2		
Sigmoid	19	6	14	2		
Length of inflamed segment (mm)^‡^	65.2 ± 21.0		85.0 ± 25.6		0.007	0.46^$^
Pericolic inflammation present^¶^—no(%)	3 (9)	7 (22)	0.097	0.47		
Area of inflammation (cm^2^)^‡^	10.7 ± 5.1		13.4 ± 5.5		0.378	0.67^$^
Colonic wall						
Maximal colonic wall thickness	11.7 ± 3.0		13.5 ± 2.9		0.060	0.58^$^
Enhancing colonic wall*—no (%)	5 (19)	11 (41)	4 (27)	5 (33)	0.698	0.46
Lymph nodes enlarged—no (%)	0 (0)	10 (31)	2 (13)	1 (6)	0.106	0.19
Size > 1 cm	0	0	0	0		
Multiple small	0	10	2	1		

^‡^Mean and standard deviation

^¶^ROI average greater than 0 Hounsfield unit

*6 CTs performed without intravenous contrast

^#^Interrater reliability is calculated as Cohen’s kappa value unless indicated otherwise

^$^Interrater reliability is calculated as intra-class correlation coefficient

**Cohen’s kappa could not be calculated because one of the radiologists rated all patients positively

Consensus: only patients that both radiologists independently agreed upon

No consensus: only patients that were designated for that outcome by one radiologist

In both groups, the predominantly affected segment was the sigmoid colon. The length of the inflamed colon segment was significantly greater in the complicated group compared to the uncomplicated group (mean 85 ± 26 mm vs. 65 ± 21 mm, respectively; *p* = 0.007). The presence of pericolic inflammation was non-significantly higher in the complicated group but the area of inflammation was comparable between groups. The maximal colonic wall thickness was non-significantly higher in the complicated group (Table 2).

#### Interrater reliability

The level of agreement between radiologists was moderate to substantial for most radiological findings. The agreement for the significant predictors for progression into complicated diverticulitis was substantial for fluid collections (ƙ 0.70) and moderate for length of inflamed colon segment (ICC 0.46). Parameters that are most frequently assessed by radiologists in daily practice yielded the highest interrater reliability levels such as extraluminal air (ƙ 0.81), free fluid (ƙ 0.68), and fluid collections (ƙ 0.70). Parameters with the lowest level of agreement were diameter of the largest diverticulum (ICC 0.42) and the presence of enlarged lymph nodes (ƙ 0.19).

## Discussion

In the present study, several predictive factors on CT for progression into complicated diverticulitis were identified. Fluid collections and to a lesser extent the length of the inflamed colon segment may serve as predictive factors on initial CT for a complicated disease course in patients who present with uncomplicated acute colonic diverticulitis. Pericolic extraluminal air was no predictive factor.

Only one previous study assessed CT imaging-based predictive factors for the progression of uncomplicated diverticulitis into complicated diverticulitis [8]. In that study, only four uncomplicated diverticulitis patients progressed into complicated diverticulitis or needed emergency surgery within 1 year. Therefore, statistical power may have been insufficient to identify predictive CT findings. Also, the type of complications was not reported. No significant predictors were identified, although their slightly longer length of the inflamed colon segment in the complicated group corresponds with the significantly longer length of the inflamed colon segment in the present study.

The present study is limited by the small number of patients with a complicated disease course of initially uncomplicated diverticulitis. This also reflects the low probability of uncomplicated diverticulitis actually progressing into complicated diverticulitis. Also, not all CTs from uncomplicated diverticulitis patients were re-evaluated but with two uncomplicated patients for each complicated patient being re-evaluated, a fair comparison could be made. Another limitation might have been that CT imaging was performed in 11 different hospitals that did not all use the same CT scans and same CT settings. To account for these differences as much as possible, two uncomplicated cases were selected from the same hospital as the complicated case. Moreover, the study duration of just over 2 years prevented technical progress of CT scanners having an influence on the study results. Another limitation is the varying level of agreement between radiologists for the radiological findings. An earlier study shows a substantial to almost perfect interrater reliability regarding the classification of acute diverticulitis with a Cohen’s kappa between 0.72 and 0.83, depending on the classification that was used [9]. Most individual CT findings appear to have a lower level of agreement between radiologists in the present study, indicating that these parameters are assessed less reliably than the classification of disease stage. The differences in specialization and years of experience between the two radiologists could also have played a role. Although, since CTs from an emergency department are likely to be evaluated by less experienced or not gastro-intestinally specialized radiologists, these differences in level of agreement should be taken into account when interpreting CT results in daily practice.

The present study identified CT findings that may predict complications in uncomplicated diverticulitis patients. It is however not clear whether these patients would not have developed complications when assigned to an antibiotic and inpatient treatment strategy. No study thus far has been able to show whether any treatment could prevent complications to develop. However, one could hypothesize that more aggressive treatment of patients at risk of developing complications could make the clinical course milder, identify, and therefore treat complications sooner, or even prevent complications. Therefore, fluid collection and a longer inflamed colon segment on initial CT imaging of a patient diagnosed with uncomplicated diverticulitis may aid in the selection of patients not suitable for outpatient treatment and treatment without antibiotics.
